# The In-Situ Mechanical Properties of Carbon Fiber/Epoxy Composite under the Electric-Current Loading

**DOI:** 10.3390/polym14204452

**Published:** 2022-10-21

**Authors:** Runtian Zhu, Xiaolu Li, Cankun Wu, Longji Du, Xusheng Du

**Affiliations:** 1 Key Laboratory for Transport Industry of Bridge Detection Reinforcement Technology, Chang’an University, Xi’an 710064, China; 2 Zhuhai Communication Group, Zhuhai 519000, China; 3 Guangdong Provincial Transport Planning and Research Center, Guangzhou 510199, China; 4 Highway Bridges National Engineering Research Center, Beijing 100088, China; 5 The Key Laboratory of Urban Security and Disaster Engineering of Ministry of Education, Beijing University of Technology, Beijing 100124, China; 6 Institute of Advanced Wear & Corrosion Resistant and Functional Materials, Jinan University, Guangzhou 510632, China

**Keywords:** carbon fiber, composites, joule heating, epoxy resin, flexural strength, interfacial property

## Abstract

The Joule heating behavior of the carbon fiber/epoxy composite (CF/EP) was studied in this work, as well as their influence on the in-situ mechanical properties of the composites and their de-icing performance. The equilibrium temperature of the CF/EP composite could be conveniently adjusted by tuning the current according to the Joule’s law. Dynamic mechanical analysis (DMA) tests indicated that the rigidity and stiffness of the fiber-reinforced composite decreased with increasing temperature, and the glass transition temperature (Tg) of the composites was around 104 °C. It was found that the flexural properties of the composites in situ, measured under the electric-current loading, depended on the current value in the range of room temperature to Tg. With increasing the loading current, either the flexural modulus or strength of CF/EP decreased gradually. Such results could be explained that the higher current loading, the larger Joule heat, led to the higher operating temperature of the composite samples and the evolution of their mechanical properties accordingly. Vickers hardness tests indicated that the micro-hardness of the composite decreased with the increase of the operating temperature, which coincided with the evolution of its flexural properties with the electric-current loading. The dependence of the failure behaviors of the CF/EP on the loading current was revealed by the analysis of their fractured surface, where micro-buckling, kinking, fiber pull-out and breakage were involved. A preliminary study indicated that less energy was consumed for the deicing of the same amount of the ice with the CF/EP composite in the case of less electric-current loading. The research on the Joule heating effect of CF/EP and their corresponding mechanical properties benefits the design and direct application of the composites under the electric-current loading.

## 1. Introduction

Due to their high specific strength, light weight, good anti-corrosion properties and chemical stability, Fiber Reinforced Polymers (FRPs) have been widely used as the important components in many fields, such as wind turbine blades, wing skins and tails in airplanes, automobiles and reinforcing structures in buildings [1,2,3,4,5].

However, low temperatures, or even ice coating is one of the service environments that occur more and more in extreme climates recently [6,7]. These lead to deviation from the designed performance of the components, and worsen the mechanical characteristics, decrease their life, and eventually endanger the safety of the whole structures. Therefore, quite a few methods have been developed to alleviate this problem by certain heating approaches.

Among the various heating methods, electric heating and deicing have been demonstrated as an effective method [8,9]. To achieve this, many designs have been proposed, such as introducing an electro-thermal layer on the surface (or embedding inside) of the components. Traditionally, additional metal heating/deicing layers had been incorporated in the material system of the components. However, such a configuration could cause some serious problems due to the failure resulting from the corrosion-expansion of the metals and/or mismatch of thermal expansion coefficients between them and FRPs [10].

Conductive carbon-based materials, including graphite, carbon fiber and carbon nanotube, could be used to replace the metals to form new electro-thermal deicing layers after the special insulation treatment [11,12,13]. Evidently, these designs will avoid the mismatch between metals and CFRPs or the electrochemical corrosion of metals. One important issue for developing the heating and/or deicing of the FRP composites is to study the effect of the temperature effect on the mechanics of FRP. The recommended service temperature of FRP composites is normally below their glass temperature (Tg). In fact, lots of work has been specifically conducted to identify the influences of high temperatures on mechanical properties of the composites. It was revealed that the tensile strength of CFRP changed little below their Tg, but drops rapidly through the glass transition phase, and finally reaches a plateau in the range from 20 to 120 °C [14]. The effect of the operating engine temperature at 120 °C on the FRP’s interlaminar shear strength, compressive modulus and compressive strength have been studied very recently [15]. With the static and dynamic three-point bending tests, the mechanical properties of CFRP exposed to temperatures ranging from −100 °C to 100 °C were evaluated as well, and they displayed relatively poor strength at the higher temperatures [16]. Li et al. investigated the mechanical properties of glass fiber-reinforced polymer exposed to temperatures ranging from 100 to 350 °C for up to 2 h [17]. Similar research was also carried out on the tensile properties of CFRP by Ashrafi et al. [18]. The results demonstrated that the mechanical properties of the CFRP composite samples were dependent on their operating temperature. The thermo-mechanical and mechanical properties of the autoclaved woven carbon fiber/epoxy composite laminate subjected to different operating temperatures (up to 180 °C) was also investigated [19]. It was found that all mechanical properties, such as compressive modulus, compressive strength and interlaminar shear strength, decreased progressively with the operating temperature. Obviously, most research work in the field is focused on the CFRP laminates under different operating temperatures, rather than the electric-current loading. Moreover, little information on the in-situ mechanical properties of the pultruded continuous carbon fiber-reinforced polymer composite under the electric-current loading is available as of yet. In order to carry out in-depth research in this field, it is crucial to clarify the relation between three aspects, i.e., mechanical properties, electric-current loading and temperature, which make it uneasy to carry out the research directly with the common instruments.

This paper aims to explore how the electric current affect the Joule heating behavior of the carbon fiber/epoxy composite (CF/EP) fabricated with the pultrusion method and their in-situ mechanical properties. The flexual properties of the CF/EP composite were measured directly under the loading of different electric currents. The failure modes of the CF/EP composite were studied as well. The dependence of the Vickers micro-hardness of the composite on the operating temperature was also studied. Additionally, the deicing performance and efficiency of the CF/EP composites under various electric currents were investigated.

## 2. Experimental Materials and Instruments

### 2.1. Experimental Materials

High-strength carbon fiber-reinforced epoxy resin used in this research was provided by Yangzhou Farber Carbon Fiber Products Co., Yangzhou, China, and continuous carbon fibers (T 300) were utilized to prepare the composite with the fiber content of ~43 wt%. The CF/EP specimens were prepared with a dimension of 10.0 mm (Width) × 2.0 mm (Thickness) × 80.0 mm (Length) for the mechanical tests, and the longitudinal direction of the specimens was manufactured along the fiber longitudinal direction of the composites.

### 2.2. Test Instrument and Method

**Figure 1:**
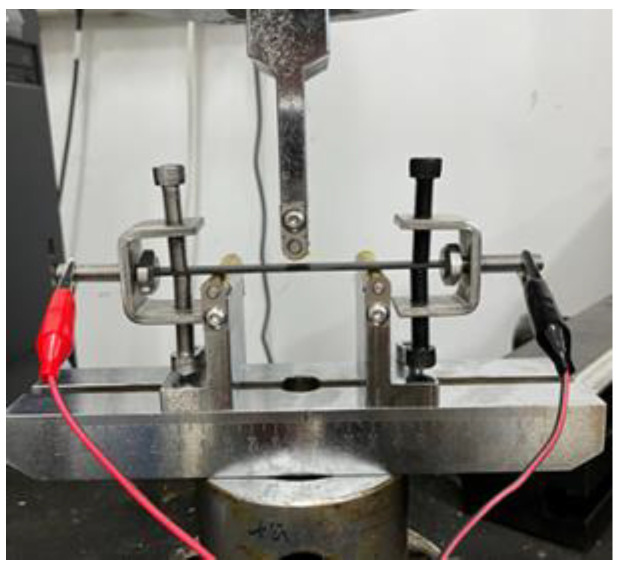
The configuration of the 3-point bending test under the electric-current loading.

The static three-point bending tests were performed on a mechanical tester (WDT, Kaiqiangli Test Instrument Co., Ltd., Shenzhen, China), according to the ASTM D7264 standard. The digital photo of the set-up to enable mechanical tests under the loading of various direct currents (supplied by Masheng MP1205D DC power supply, Dongguan, China) was provided as Figure 1, where both the cross-section faces of the CF/EP plates were polished and clamped with steel fixtures, which also acted as electrodes to reduce the contact resistance. For insulation, PI films of 25 μm were applied on both the indenter tip and the surfaces of the two supporting frames of the fixture for the bending test. During the tests, the electric currents 3 A, 4 A and 5 A were selected to be loaded onto the specimens, and the temperature was monitored with an infrared thermal imager (Xinst HT-19, Dongguan, China). For each current loading, at least three specimens were tested, and all specimens were kept under the loading of the electric current for more than 3 min, in order to achieve a stable temperature before starting the tests. All the experiments were conducted in a quasi-static regime, and the load-displacement curves measured from the 3-point bending tests were then transferred into the flexural stress-strain behaviors based on the measured dimensions of the CF/EP specimens. The cross-head speed was set to be 2 mm/min for all the bending tests. The following equation is adopted to calculate the flexural strength *σ*

(1) $$\sigma =\frac{\text{3}PL}{2bh^{2}}$$

where *σ* is the flexural strength, *P* is the load applied at the middle of the span, *L* is the support span (64 mm) and b and h are the width (10 mm) and thickness (2.0 mm) of specimens, respectively.

The electric conductivity of the composite was measured in two-probe mode, and the current was loaded along the longitude of CFs in the CF/EP samples. For the deicing tests, the same amount of water was directly frozen on the surface of the CF/EP specimens in a refrigerator. After the loading of different electric currents, the time for the total melting of the ice was recorded and compared. The hardness of CF/EP sample was measured on a Vickers hardness tester under HV = 0.1, and the temperature of the sample was controlled by the smooth aluminum plate heating stage whose temperature could be well adjusted in the range of room temperature to 300 °C. Dynamic mechanical analysis (DMA) of the sample was performed on a DMA1 instrument (METTLER TOLEDO, Columbus, OH, USA). ZEISS ultra plus SEM (Germany) was used to observe the fractured surface of composite samples, which was sputter-coated with a thin Au layer before the observation.

## 3. Results and Discussion

### 3.1. Electric Heating Behavior

**Figure 2:**
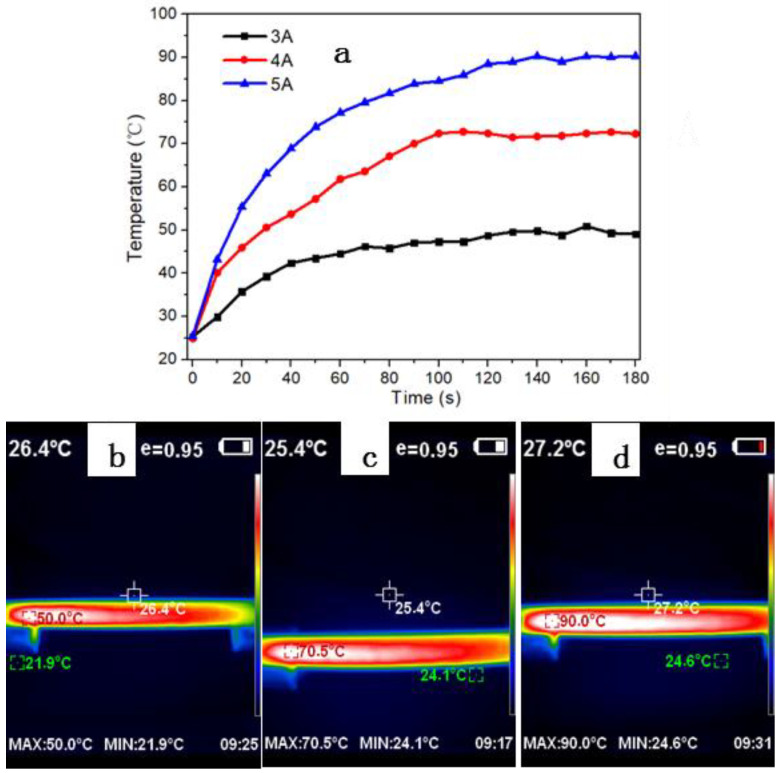
Infrared thermal imaging of CF/EP plates with different conduction currents: (**a**) The temperature response to the current loading time; the typical infrared photograph of the samples under the current loading of (**b**) 3 A, (**c**) 4 A and (**d**) 5 A.

Before the investigation of the dependence of the electrical current on the in-situ mechanical properties of the CF/EP plates, the Joule heating behavior at different electric-current loading was studied, including the temperature response rate and the equilibrium surface temperature of the carbon fiber composites. The current was loaded along the longitude direction of carbon fibers in the CF/EP plate. Such a circuit would leave more heating area exposed to the environment for their electric-heating applications. The electrical conductivity of the CF/EP plate was tested to be 10.5 S/cm, and changed little with the different electric currents. This was close to the previously reported conductivity value measured in the in-plane and fiber longitude direction of the unidirectional CF/EP laminate [20]. As shown in Figure 2a, the surface temperature response rate of the CF/EP was very fast, and it increased quickly within the first stage before reaching an equilibrium surface temperature. Obviously, the temperature response rate increases with the increased loading currents. Within the first 10 s, the elevated temperature under 5 A was about 3.6 times that of under 3A. However, the time required to reach the surface equilibrium temperature seemed to always be within roughly 2 min, despite the different electric current (Figure 2a). This was ascribed to the enhanced electric heating rate under larger electric-current loading, based on Joule’s law (thermal power = I^2^R, where R was the resistance). These results meant that both the temperature response rate and the surface equilibrium temperature of the CF/EP could be conveniently adjusted by tuning the electric current loaded on the specimens. The typical infrared photographs of the CF/EP under the current loading of 3 A, 4 A and 5 A were shown as Figure 2b–d, respectively. It was noted that the equilibrium surface temperature at 3 A is larger than that of CF/EP laminate under the same current loading (~37 °C) [21]—this could be due to the different geometry and distribution of CF in the matrix of the samples. The pultruded CF/EP with such a good Joule heating behavior under a small electric current was in favor of its potential multi-applications, including de-icing [6,11,12,13,22,23].

### 3.2. Dynamic Mechanical Performance of the Fiber Reinforced Composites

**Figure 3:**
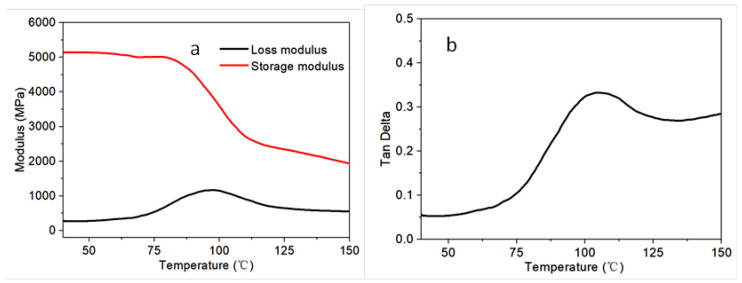
Dynamic mechanical property of the CF/EP composite: The dependence of storage modulus and loss modulus (**a**) and Tan Delta (**b**) on the temperature.

In order to study the mechanical properties of the sample as a function of temperature, the dynamic mechanical analysis (DMA) was first utilized to measure their storage modulus, loss modulus and damping factor (Tan Delta). As shown in Figure 3, the glass transition region for the CF/EP composite was from about 75 to 104 °C, with a glass temperature (Tg) of 104 °C as a local maximum of the Tan Delta. The loss modulus of CF/EP composite could identify its capability to dissipate energy applied on it, and changed little with a temperature before 60 °C, and an obvious increasing of it was observed in the temperature range of 60 and 100 °C (Figure 3a). This could be mainly due to the enhanced mobility of the polymeric chains of the matrix at high temperatures, in consideration of the near-independence of the mechanical properties of CFs on the temperature in the testing temperature range. The damping factor (Tan Delta) also displayed a similar trend to that of the loss modulus (Figure 3b).

### 3.3. Electric Current Loading Effect on the In Situ Flexural Properties of the CF/EP Composites

**Figure 4:**
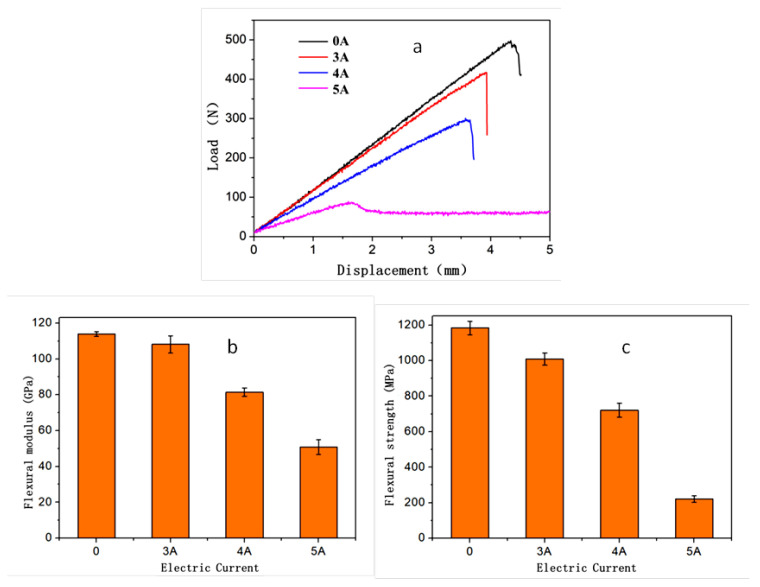
The three-point bending results of the CF/EP composites under the loading of different electric currents from 0 to 5 A. (**a**) Representative force-displacement curves; (**b**) flexural modulus; and (**c**) flexural strength.

Theoretically, the interfacial properties of carbon fibers in the CF/EP composites may deteriorate due to the Joule heating with regards to the dependence of mechanical properties of the epoxy matrix on the temperature, according to the DMA test results (Figure 3), as well as the incomparable thermal expansion of epoxy matrix and carbon fibers. Therefore, the flexural properties of CF/EP composite plates were tested to evaluate the electric-current loading effect on their mechanical properties. The typical flexural load–displacement curve of the sample under each current was also shown in Figure 4a. Evidently, the curves for all the specimens exhibited a linear elastic regime, followed by a stress drop, which meant that fracture or failure occurred. Under the currents of 0, 3 A and 4 A, the stress drop was violent and catastrophic. In contrast, it came to be mild once the loading current was increased to 5 A, and the stress decreased very slowly before reaching a plateau. Both the flexural modulus and strength of the composite plate under the current loading of 0, 3 A, 4 A and 5 A were calculated and presented in Figure 4b,c, respectively. As shown in Figure 4b, the flexural modulus of the CF/EP composite under 5 A (whose equilibrium temperature is about 90 °C, according to Figure 2) was less than half of those under 0 and 3 A, and its flexural strength was around only 1/6 of that of the sample without any current loading.

The evolution of the mechanical properties of the CF/EP composites under different electric-current loading could be attributed to the increasing temperature of the samples originating from Joule heating. Under the current loading of 5 A, the equilibrium temperature of the composite was around 90 °C, which was located in the glass transition region of the composite, and near to the rubbery state. During the bending test of the CF/EP composite plate samples, large shear deformations took place in the matrix, together with small tensile/compressive deformation. Meanwhile, the bent carbon fibers in CF/EP composite had relatively small longitudinal deformation due to their 1D morphology, therefore contributing less to the flexural modulus of the CF/EP composite. However, when the epoxy matrix of the composite was in its glassy regime (T < 60 °C, 0 A and 3 A), the shear stress loaded on the epoxy matrix could be transferred effectively to CFs, and the two load-displacement curves of the CF/EP composite under each electric current almost approached each other (Figure 4a).

**Figure 5:**
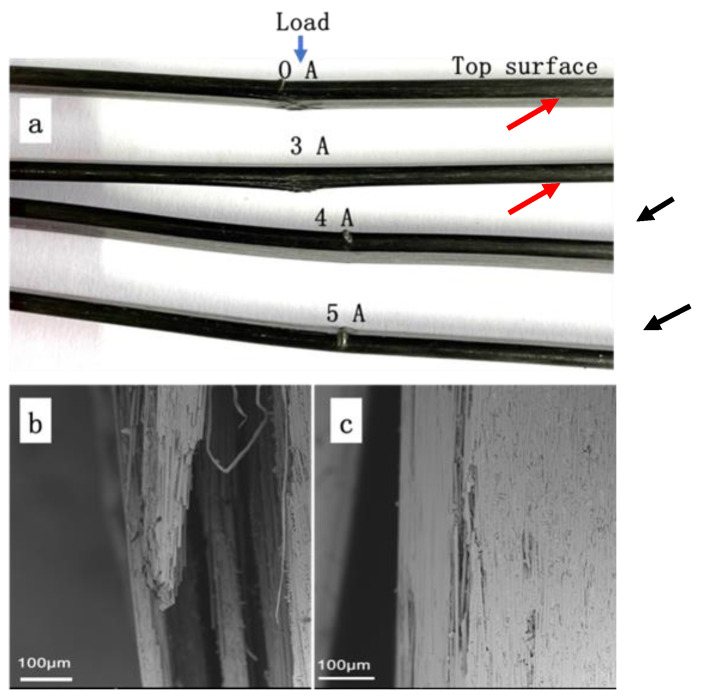
The photographs of the CF/EP composite plates after the 3-point bending tests. (**a**) Digital photographs of side view of the composites loaded with different electric current; SEM image of the bottom layer of the CF/EP composite under (**b**) 3 A and (**c**) 5 A.

**Figure 6:**
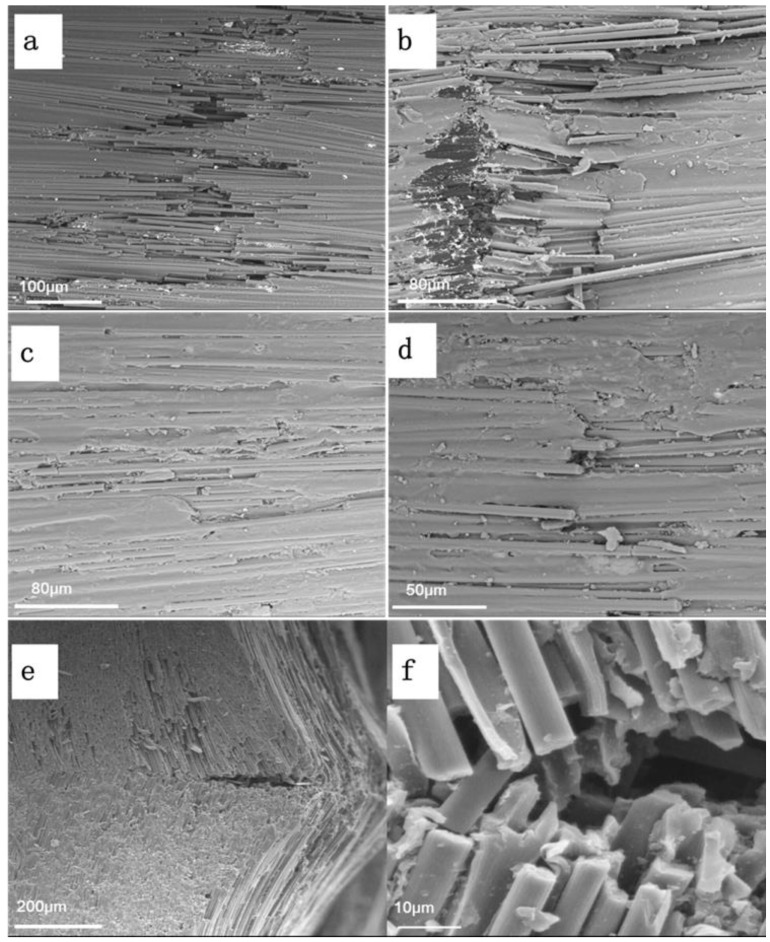
SEM observation of the CF/EP specimens under different current loading (top view): (**a**) bottom layer and (**b**) upper layer of the specimens under 3 A; (**c**) bottom layer and (**d**) upper layer of the specimens under 5 A; and (**e**,**f**) the side view of the buckling area on the upper layer of the specimens under 5 A.

In order to study the failure mechanism of C/EP composite after the 3-point bending tests under different electric current loading, the morphologies of the failed sample were observed and analyzed. Figure 5a showed digital photographs of the specimens. The specimens under 0 A and 3 A exhibited obvious failure by delaminating at bottom surfaces, as highlighted with red arrows in Figure 5a, indicating that the major crack was localized in the mid-section, perpendicular to the fiber direction. Such serious delamination and fiber breakage occurred in the cases of the composites under both 0 A under 3 A, and the typical fractography was given as Figure 5b. In contrast, both the upper and bottom layers of the composites with the loading of a larger current (4 A and 5 A) under the indenter remained visually intact (Figure 5a). These results meant that the minor fiber breakage and debonding from the polymer matrix took place in the composite, and their characteristic fractography was shown as Figure 5c. This could originate from the failures of part of epoxy resin due to the localized deformation, which resulted in several inter-fiber cracks. Being close to the Tg, the epoxy resin matrix likely worked as an elastomer under this current loading, and therefore most deformation (except the tensile deformation located on the bottom layer of the sample) could be recovered after the bending test, leading to no significant failure and deformation. On the other hand, under the current loading of 0 A and 3 A, the temperature of the CF/EP sample was far less than its Tg, leading to its typical behavior of the brittle thermosetting epoxy matrix. Therefore, evident matrix/CF failure and delamination occurred on the bottom layer of the samples. A further top view of the bottom layer of the CF/EP sample under 3 A was shown in Figure 6a, where the fiber breakage, pull-out and extensive fiber-matrix debonding were observed. By contrast, only insignificant fiber breakage or debonding from the matrix were found for the sample under 5 A (Figure 6c), and most carbon fibers stayed intact, suggesting the local plastic deformation of the resin matrix.

For the sample under small current of 3 A and without current loading (0 A, at room temperature (25 °C)), kink bands appeared on the upper layer of samples near the indenter. In the kink bands, plenty of bundle cracking could be observed readily with SEM (Figure 6b), although they were not so evident as their counterpart under naked eyes (Figure 5a). When the electric current increased to 5 A, the fractography (Figure 6d) similar to that of the bottom layer surface of the sample (Figure 6c) could be observed on the upper layer, where epoxy matrix deformation and inter-fiber cracks could be seen. However, micro-buckling could be observed on the upper layer at the site near the indenter, as highlighted by black arrows in Figure 5a. Further enlarged SEM images of the specific position (Figure 6e–f) indicated that the elastic buckling led to the extensive breakage of fibers due to the great deformation under larger electric-current loading, which was unable to be recovered after unloading.

### 3.4. Hardness of the CF/EP Composites under Different Temperature

**Figure 7:**
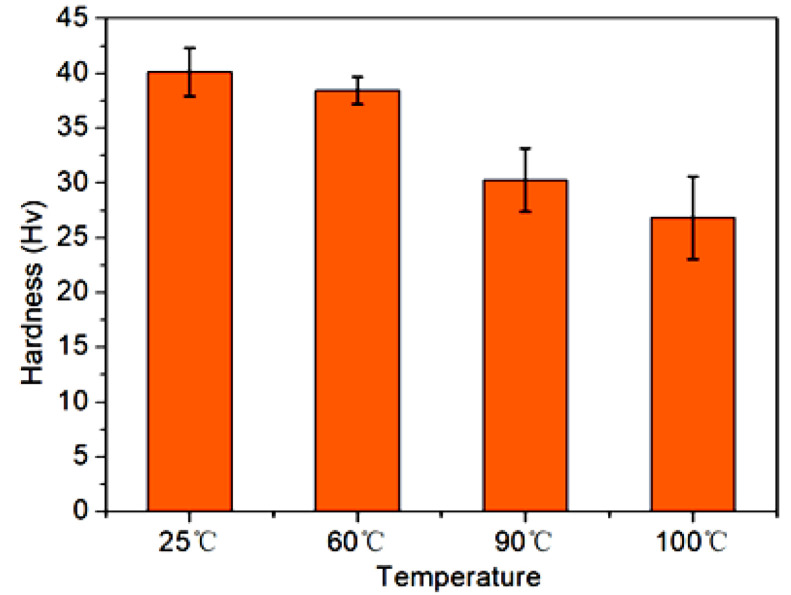
The dependence of the in-situ micro-hardness of the CF/EP composite on the temperature.

The in-situ micro-hardness values of the CF/EP composites at different operating temperature were measured in consideration of the high equilibrium temperature of the samples resulting from the Joule heating during the electric-current loading (Figure 2). As shown in Figure 7, in comparison with that measured at room temperature (25 °C), the micro-hardness of CF/EP samples decreased a little at the operating temperature of 60 °C, which could be achieved by the loading of certain current between 3–4 A, according to the relationship between the equilibrium temperature and the conduction current of the composites (Figure 2). With further increasing the operating temperature to 90 °C or above (>5 A), the hardness of the fiber composites decreased evidently. At 100 °C, the hardness of the CF/EP composite was greatly reduced, and was about 35% less than that measured at 25 °C. This phenomenon should be also ascribed to the dependence of the mechanic characteristics of epoxy matrix on the operating temperature, and was inconsistent with the evolution of the flexural properties of the CF/EP composites under various conduction current loading (Figure 4). Additionally, the interface between the epoxy matrix and CF at a high operating temperature should also deteriorate due to the incomparable thermal expansion coefficient between the polymer matrix and the inorganic carbon materials. This would also contribute to the reduction in the hardness of the CF/EP composites under high temperatures.

### 3.5. Deicing Performance

**Figure 8:**
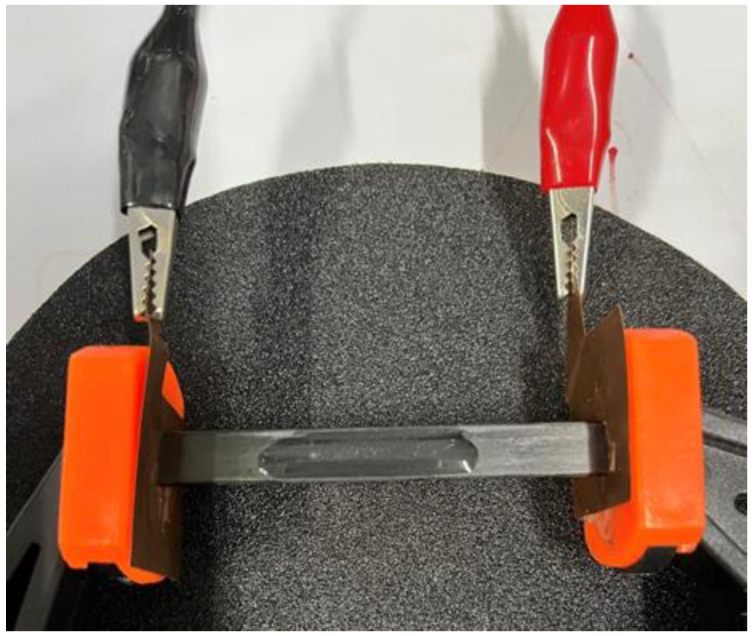
The experimental set-up for the deicing test of the CF/EP composite plate.

Traditionally metallic heating elements were employed for the Joule heating applications in aeronautics, electric ice protection systems, etc. [24]. In this work, the structural composite itself played a similar role, but avoided the additional incorporation of the materials in the composites. The aforementioned results had demonstrated that the in-plane direction of the CF/EP showed its Joule heating behavior under various electric-current loading and their effect on the mechanical properties of the composite. In order to retain satisfying mechanical properties of the CF/EP plates, 3 A is firstly applied to the plates to evaluate its electric-heating and deicing performance. The typical test for the deicing performance of the CF/EP plates was shown as Figure 8, and the same amount of ice layer was fabricated on the surface of the plates in a refrigerator. Table 1 showed the time required for the ice layer completely melting into water during the deicing process. It indicated that the required time for deicing decreased with the increase of the electric-current loading. 60 s was needed for the CF/EP plate to completely melt the ice layer into water under the current loading of 3 A. As expected, the deicing process could be easily shortened by loading a larger electric current. Under 5 A, the corresponding time for completely melting the ice into water was decreased to be 40 s (Table 1). Generally, the required energy was needed to input in the deicing process to melt the ice in a certain period of time. The larger the electric current being loaded to the CF/EP plate, the more heat energy was generated for the deicing process. It should be noted that under larger current loading, the output Joule’s energy should not be as much as expected based on the Joule’s law (Q = W = UIt = I^2^Rt). The input power of CF/EP was calculated to be 756 J at 3 A, while it was 1400 J under 5 A, indicating that less energy was required for the deicing process under the small current loading. This could be due to more heat loss or dissipation into the surrounding environment in the case of larger current loading.

## 4. Conclusions

In this paper, the Joule heating behavior of the pultruded epoxy composite reinforced with the continuous carbon fibers was studied, as well as their influence on the in-situ mechanical properties of the composites and their de-icing performance. The conclusions were as follows:

1. The equilibrium temperature of the CF/EP composite could be conveniently adjusted by tuning the direct current loading according to the Joule’s law. Under the 3-point bending test, the temperature of the sample could reach an equilibrium temperature of 91 °C under an electric-current of 5 A within 3 min.
2. Dynamic mechanical analysis (DMA) tests indicated that the Tg of the CF/EP composite was around 104 °C, and the stiffness of the fiber reinforced composite decreased with increasing temperature.
3. With increasing the loading current, both the flexural modulus or strength of CF/EP composite decreased. At 5 A, its flexural strength was around 1/6 of that without any current loading—this was caused by the increased temperature with the loading current.
4. Vickers hardness tests demonstrated similar dependence of micro-hardness of CF/EP on the operating temperature. The hardness obtained at 100 °C was about 2/3 of that measured under 25 °C.
5. The failure behaviors of the CF/EP composite also depended on the loading current. It was found that micro-buckling, kinking, fiber pull-out and breakage were involved in the fracture process.
6. The deicing process involved with the CF/EP composite could be easily shortened by loading a larger electric current. However, the deicing energy consumed at a lesser electric current could be reduced greatly.

## Figures and Tables

**Table 1:** The deicing behavior of the CF/EP composite under different current loading.

Electric Current (A)	Melting Time (s)	Joule Heat Energy (J)
3	60	756
5	40	1400

## Data Availability

Not applicable.
